# Different Biosynthesis Patterns among Flavonoid 3-glycosides with Distinct Effects on Accumulation of Other Flavonoid Metabolites in Pears (*Pyrus bretschneideri* Rehd.)

**DOI:** 10.1371/journal.pone.0091945

**Published:** 2014-03-17

**Authors:** Rui Zhai, Xiao-Ting Liu, Wen-Ting Feng, Sha-Sha Chen, Ling-Fei Xu, Zhi-Gang Wang, Jiang-Li Zhang, Peng-Min Li, Feng-Wang Ma

**Affiliations:** College of Horticulture, Northwest A&F University, Yangling, Shaanxi, China; Key Laboratory of Horticultural Plant Biology (MOE), China

## Abstract

Flavonoid biosynthesis profile was clarified by fruit bagging and re-exposure treatments in the green Chinese pear ‘Zaosu’ (*Pyrus bretschneideri* Rehd.) and its red mutant ‘Red Zaosu’. Two distinct biosynthesis patterns of flavonoid 3-glycosides were found in ‘Zaosu’ pear. By comparison with ‘Red Zaosu’, the biosynthesis of flavonoid 3-galactosides and flavonoid 3-arabinosides were inhibited by bagging and these compounds only re-accumulated to a small degree in the fruit peel of ‘Zaosu’ after the bags were removed. In contrast, the biosynthesis of flavonoid 3-gluctosides and flavonoid 3-rutinosides was reduced by bagging and then increased when the fruits were re-exposed to sunlight. A combination of correlation, multicollinearity test and partial-correlation analyses among major flavonoid metabolites indicated that biosynthesis of each phenolic compound was independent in ‘Zaosu’ pear, except for the positive correlation between flavonoid 3-rutincosides and flavanols. In contrast with the green pear cultivar, almost all phenolic compounds in the red mutant had similar biosynthesis patterns except for arbutin. However, only the biosynthesis of flavonoid 3-galactosides was relatively independent and strongly affected the synthesis of the other phenolic compounds. Therefore, we propose a hypothesis that the strong accumulation of flavonoid 3-galactosides stimulated the biosynthesis of other flavonoid compounds in the red mutant and, therefore, caused systemic variation of flavonoid biosynthesis profiles between ‘Zaosu’ and its red mutant. This hypothesis had been further demonstrated by the enzyme activity of UFGT, and transcript levels of flavonoid biosynthetic genes and been well tested by a stepwise linear regression forecasting model. The gene that encodes flavonoid 3-galacosyltransferase was also identified and isolated from the pear genome.

## Introduction

Flavonoid secondary metabolites are associated with a multitude of biological functions. The pigments that color fruit peels are anthocyanins and proanthocyanins which are flavonoid metabolites [1]. The colorless flavonoid compounds (e.g. flavanols) are hidden by the ubiquitous green of the chlorophylls in epidermal cells of leaves, buds or fruits, but they, as well as anthocyanins, make significant contributions to resistance to UV light and pathogens [2], [3]. With their antioxidant activity, these flavonoids also have a health benefit to humans [2].

The Chinese pear ‘Red Zaosu’ originated as a spontaneous mutation from ‘Zaosu’ (*Pyrus bretschneideri Rehd.*). The young fruit of ‘Red Zaosu’ is dark red, but then its color intensity continuously decreases accompanied with the appearance of green stripes on its surface during the maturation. In ‘Red Zaosu’ fruit, growth parameters and fruiting characters are similar with those in ‘Zaosu’ [4].

**Figure 1:**
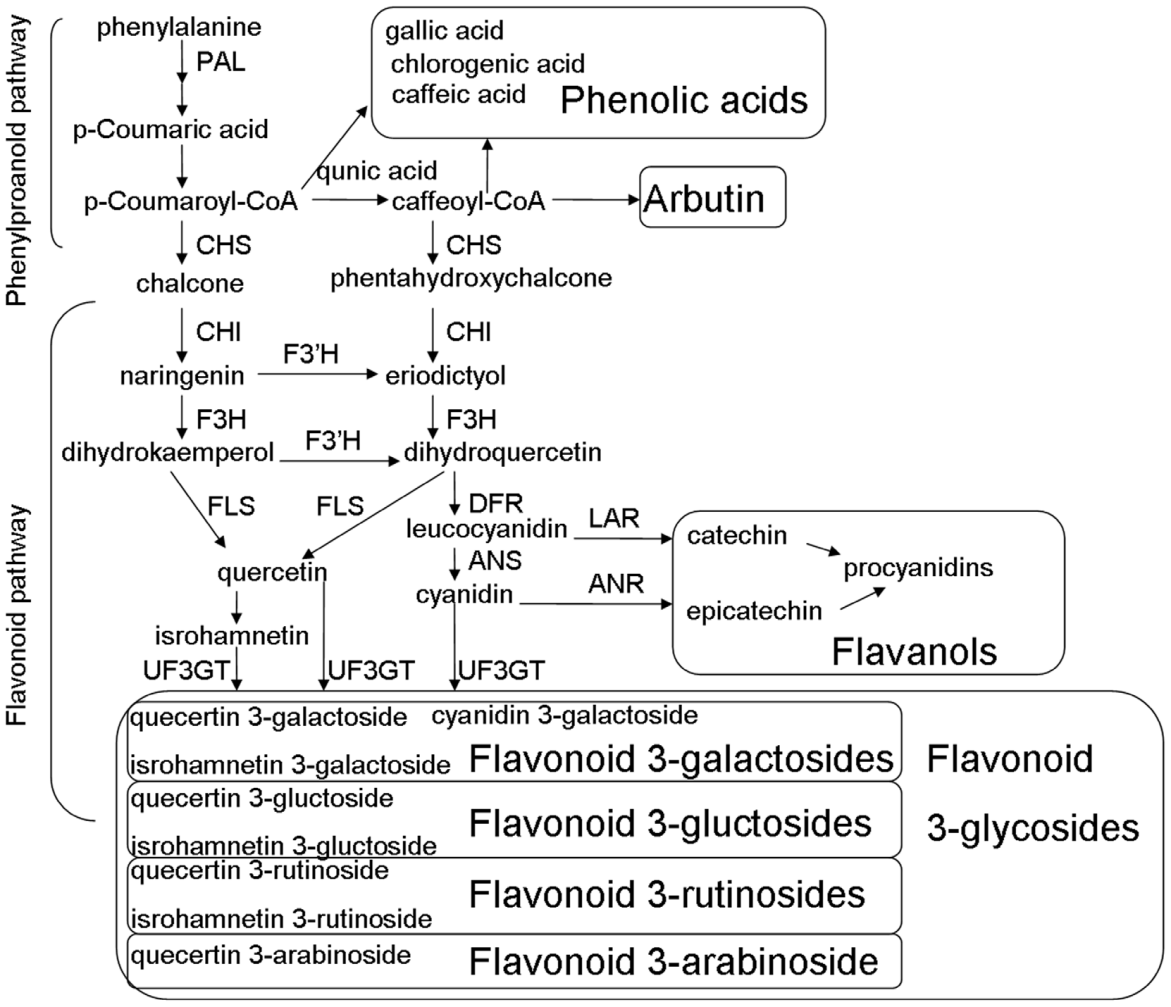
Flavonoid pathway in pear.

The flavonoid pathway in pear fruits is well established (Fig. 1) [5]. Phenylalanine is the precursor for the synthesis of many polyphenols, and is enzymatically converted to flavonoids via many steps. The enzymes involved in the pathway include phenylalanine ammonialyse (PAL), chalone synthase (CHS), flavanone 3-hydroxylase (F3H), dihydroflavonols 4-reductase (DFR), anthocyanin reductase (ANR), anthocyanin synthase (ANS), flavonol synthase (FLS), and UDP-glucose: flavonoid 3-glucosyltransferase (UFGT) [5], [6]. Changes in the transcriptional levels of flavonoid biosynthetic genes could cause systematic variation in flavonoid biosynthesis. The silencing of *MdANS* in a transgenic red *Malus* hybrid significantly reduced anthocyanin content accompanied by rises in flavanols and flavonols [7]. Transcriptional factors in the regulation of flavonoid biosynthesis in pear fruits, *PyMYB10* and *PcMYB10* were isolated via homology with apple sequences [8]–[10]. In the cultivar ‘Early Red Doyenne du Comice’ and its green variant strain, most of the structural genes were up-regulated in the red cultivar during fruit development, but they were not the key factors controlling the red/green color mutant [11]. The high methylation level of the promoter of *PcMYB10*, which caused low expression levels of *PyUFGT*, was associated with the formation of the green-skinned sport in the ‘Max Red Bartlett’ pear [10].

Fruit bagging can provide ideal fruits for studying flavonoid biosynthesis, by ensuring fruit of similar developmental stage and physiological condition [12]. Bagging treatments can significantly inhibit the biosynthesis of most flavonoid compounds in apple peels. Compared with unbagged apple fruits, the bagged ones contain less anthocyanins, procyanidins, quercetin glycosides, and simple phenols in their peels [3], [ 13], [14]. The effect of fruit bagging differs among cultivars [3]. In ‘Fuji’ apple, anthocyanins gradually accumulate after the bag removal and the anthocyanin content was the highest when the bags had been removed for 20 days [15]. In red Chinese sand pears, the anthocyanins accumulated rapidly for more than 10 days after the bag was removed, then the rate of accumulation decreased as the fruits matured [16]. Although the synthesis pattern of anthocyanins has been studied by bagging and re-exposure treatments in pears and apples, the synthesis patterns of flavonols, flavanols and phenolic acids are still unclear.

In the present study, we studied flavonoid biosynthesis in ‘Zaosu’ and ‘Red Zaosu’ in terms of metabolites and gene expression. We identified two UF3GT genes related to different flavonoid 3-glycoside biosynthesis patterns. Interrelationships in the biosynthesis of different flavonoid compounds were studied by a combination of correlation and partial-correlation analyses. Moreover, we built a forecasting model based on stepwise linear regression and analyzed the enzyme activity of UF3GT and the expression patterns of flavonoid biosynthetic genes to confirm these interrelationships.

## Materials and Methods

### Plant materials

The Chinese pear cultivars ‘Zaosu’ (*Pyrus bretschneideri* Rehd.) and its red mutant ‘Red Zaosu’, the fruit of which matured in end of July, were used for materials. The trees were 5-year-old on *Pyrus betulaefolia* Bunge rootstocks which grew in a commercial plantation of Dali County (lat: 34.8542, lng: 109.8378; elevation: 368 m), Shaanxi, China. No specific permissions are required for this location. In this study, no endangered or protected species were involved.

Considering the effects of the different transcriptional levels of flavonoid biosynthetic genes during the fruits maturation, we extended the duration of re-exposure time from less a month in previous studies [13], [17] to almost two months. In early May 2012, about 30 well exposed fruits per tree were bagged for each treatment. About 30 days later, when the color of bagged fruits turned to light yellow without any redness on its surface, we started to remove bags every 8 days until the fruits matured. Eight different re-exposure duration treatments were used: 0 days (bagged fruits), 8 days, 16 days, 24 days, 32 days, 40 days, 48 days and 56 days. Non-bagged fruits were treated as the control. For each treatment, 5 replications (i.e. 5 trees) were used. To ensure the similar developmental stages, sizes and physiological conditions of the fruit, all the mature fruits (bagged,re-exposed and unbagged) were collected on the same day (24 July, 2012). For each tree replication, random samples of 20 fruit were taken. The bagged fruits were harvested without taking off the bags to avoid exposure to light before sample collections. Fruits in different natural developmental stage (25 days, 40 days, 55 days, 70 days, 85 days and 100 days after flower full bloom) were also obtained. Fruit peels (about 1 mm thick) were collected with a peeler. The samples were immediately frozen in liquid nitrogen and stored at −80°C until analysis.

### Fruit color measurement

Fruit skin color was measured on the most colored part of fruit using a colorimeter (CR-400, Minolta, Japan), which provided CIE L*, a*, and b* value. L* represents the relative lightness of color with a range from 0 to 100, being small for dark color and large for light color. Both a* and b* scales extend from −60 to 60. Negative a* value indicates greenness and positive for redness, while b* is negative for blueness and positive for yellowness [18]. For this study, we used a* value to evaluate the redness/greenness on fruit peels.

### Phenolic compounds analysis

The extraction and analysis of phenolic compounds were carried out as described by Zhang et al. [19]. Briefly, the phenolics were extracted with 70% methanol containing 2% formic acid at 0–4°C.The supernatant was filtered through a 0.45 μm syringe filter prior to HPLC analysis.

Phenolic compounds were analyzed using a HP1200 Liquid Chromatograph equipped with a diode array detector (Agilent Technology, Palo Alto, CA, USA). The Inertsil ODS-3 column (5.0 μm particle size, 4.6 mm×250mm, GL Sciences Inc., Tokyo, Japan) was used in the separation, preceded by an Inertsil ODS-3 Guard Column (5.0 μm, 4.0 mm×10 mm). Solvent A consisted of 10% formic acid (11.36% 88% formic acid) dissolved in water and solvent B was 10% formic acid and 1.36% water (11.36%88% formic acid) in acetonitrile (HPLC grade, purity: 99.9%). The gradient was 95% A (0 min), 85% A (25 min), 78% A (42 min), 64% A (60 min), and 95% A (65 min). Post-run-time was 10 min. Flow rate was 1.0 mL/min at 30°C. Simultaneous monitoring was performed at 280 nm for catechin, epicatechin, procyanidin B1, procyanidin B2, arbutin, and gallic acid; 320 nm for chlorogenic acid, caffeic acid, 365 nm for quercetin-3-galactoside, quercetin-3-glucoside, quercetin-3-arabinoside, quercetin-3-rutinoside, isrohamnetin-3-galactoside, isrohamnetin-3-gluctoside, isrohamnetin-3-rutinoside and 520 nm for cyanidin-3-galactoside, respectively. Peaks were identified by comparison of retention time and UV spectra with authentic standards. The concentration of individual phenolic compounds was determined based on peak area and calibration curves derived from corresponding authentic phenolic compounds. All the phenolic standards were obtained from SigmaAldrich (St. Louis, MO, USA), Extrasynthese (Genay Cedex, France), and AApin Chemicals (Abingdon, Oxon, UK).

### Identification of candidate UFGT genes, phylogeny analysis and cDNA cloning

The identification and analysis of candidate genes from genome database were carried out as described by Li et al. [20]. Candidate UF3GT genes were identified by performing blastn analysis against the Pear Genome Database from Centre of Pear Engineering Technology Research, Nanjing Agricultural University, China (http://peargenome.njau.edu.cn/default.asp?d=4&m=2) [21] using *MdUFGT1* (AF117267.1) and *PcUFGT* (KC460398.1) in GenBank as queries and an E-value of 1.00E-09 as the threshold. The putative candidate gene sequences were retrieved from the Pear Genome Database: http://peargenome.njau.edu.cn/default.asp?d=4&m=2/. The corresponding sequences of candidate genes were then used for a blastn search against the Pear EST database from Centre of Pear Engineering Technology Research, Nanjing Agricultural University, China (http://peargenome.njau.edu.cn/default.asp?d=4&m=2) to confirm that each predicted gene was expressed in the pear transcriptome when there is a high similarity EST sequence (score >300 bp, and identity >99%). Two putative candidate UF3GT genes, which were named as *PyUFGT1* and *PyUFGT2*, were screened for expression analysis. The full-length *PbUFGT1* cDNA was isolated by using the primer set (forward, 5′-ATGGCACCGCCGCCGCC-3′, and reverse, 5′- CTATGCTTTCTTGGATCCTGATATA -3′) designed according to its predicted CDs (from Coding DNA Sequence) sequence data in the Pear Genome Database (http://peargenome.njau.edu.cn/default.asp?d=4&m=2). Phylogenetic analysis of *Malus* and *Pyrus* amino acid sequences was performed using maximum likelihood (http://www.phylogeny.fr).

### Total RNA extraction and RT-PCR

Total RNA was extracted by using SDS-phenol method as described by Fonseca et al. [22]. The RNA concentration and quality were detected by UV spectrophotometry and by running on a 1.2% agar/EB gel.

One μg of total RNA was used for reverse-transcription to cDNA with the PrimeScript RT reagent Kit with gDNA Eraser (TaKaRa, Dalian, China). Every qRT-PCR was performed in 3 replicates on an Icycler iQ5 (BiRad) with the SYBR Premix Ex Taq II (TaKaRa, Dalian, China) according to the instruction manual. Data were analyzed by iQ5 2.0 software (BioRad) using the ddCT algorithm. The primers for *Actin*, *PbF3H*, *PbDFR*, *PbMYB10*, *PbCHS*, *PbANR*, *PbANS*, and *PbUFGT2* were the same as described by Zhang et al. [23]. Primers used for *PbUFGT1* were: forward, 5′-GGACACTATCGGAACTCAAGG-3′, and reverse, 5′- AGGTCGAGTTCTTCGAAACAG-3′.

### Enzyme measurement of UFGT

One gram of frozen tissue was homogenized using a pestle and mortar in 5% PVPP and 2.4 ml 100 mM Tris-HCl buffer (pH 8.0) containing 14 mM β-mercaptoethanol, 5 mM DTT, 2 mM EDTA, 15 mM MgCl2, 0.5% Triton X-100, 10% glycerol, and 1% BSA. The homogenate was centrifuged at 14,000 g for 20 min at 4°C. The supernatant was applied to a PD10column equilibrated with the elution buffer containing 100 mM Tris-HCl buffer (pH 8.0) and 2 mM DTT. Once the dissolved precipitate was completely absorbed onto the column, the protein was eluted with 2 mL elution buffer.

UFGT (EC 2.4.1.91): was assayed in a modification of the buffer system reported by Do et al. (1995). In a final assay volume of 200 μL, the reaction conditions were 100 mM buffer (Tris-HCl, pH 8.0), 250 mM MgCl2, 2 mM dithiothreitol, 0.9 mM UDP-glucose(or galactose), 100 μM quercetin or cyanidin chloride, and 100 μL enzyme extract. The reaction mixture was incubated at 37 °C for 10 min and extracted twice with ethylacetate. The ethylacetate was then evaporated completely. The reaction product was dissolved with methanol and analyzed by HPLC at 525 nm or 365 nm.

### Statistical analysis

Analysis of variance and significant difference tests were conducted to identify differences among means by one-way ANOVA (with Turkey's HSD test). Correlation and partial-Correlation analyses were conducted by determining the Pearson product moment correlation and its level of probability. A multicollinearity test was conducted by the Variance Inflation Factor (VIF). Stepwise linear regression analysis (SLRA) was applied to select the most suitable variables from major phenolic compounds (arbutin, phenolic acids, flavanols, flavonoid 3-galactosides, flavonoid 3-gluctosides, flavonoid 3-rutinosides, flavonoid 3-arabinosides) to forecast the hypothetical changes of flavonoid profiles. Statistical analyses and SLRA were conducted using SPSS 16.0 (SPSS, Chicago, IL, USA).

## Results

### Red color development and changes in anthocyanin levels

**Figure 2:**
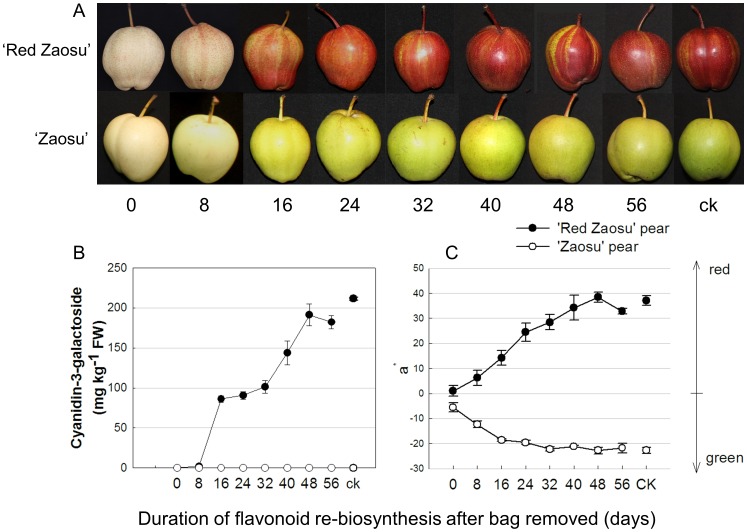
Gradually changed color of the fruits. The fruits were showed in graph A, the anthocyanidin concentrations were showed in graph B and the color data in pear fruit peel were showed in graph C. ‘a*’ value represents peel color from greenness (negative value) to redness (positive value). The treatments of re-exposed to sunlight for 0 day represented bagged fruits, natural fruits were used as controls. Error bars are SE for 5 replicates.

Fruit bagging significantly affected color parameters. Natural ‘Red Zaosu’ fruits presented the highest values for red color (component ‘a*’ after Minolta colorimeter analysis). The ‘a*’ values of bagged fruits was almost 0 in the two cultivars, which indicated no red pigment in their skins. The re-exposure fruits of ‘Red Zaosu’ had increasing ‘a*’ values from 8 days to 56 days, coincident with the gradually changed colors from light yellow to red. With the same treatments, the light yellow ‘Zaosu’ fruits gradually turned to green. The anthocyanin level corresponded to the changing patterns of ‘a*’ values in ‘Red Zaosu’ pear, and we did not find any anthocyanins in bagged, re-exposed or natural fruits of ‘Zaosu’ pear (Fig. 2).

### Biosynthesis patterns of phenolic compounds

**Figure 3:**
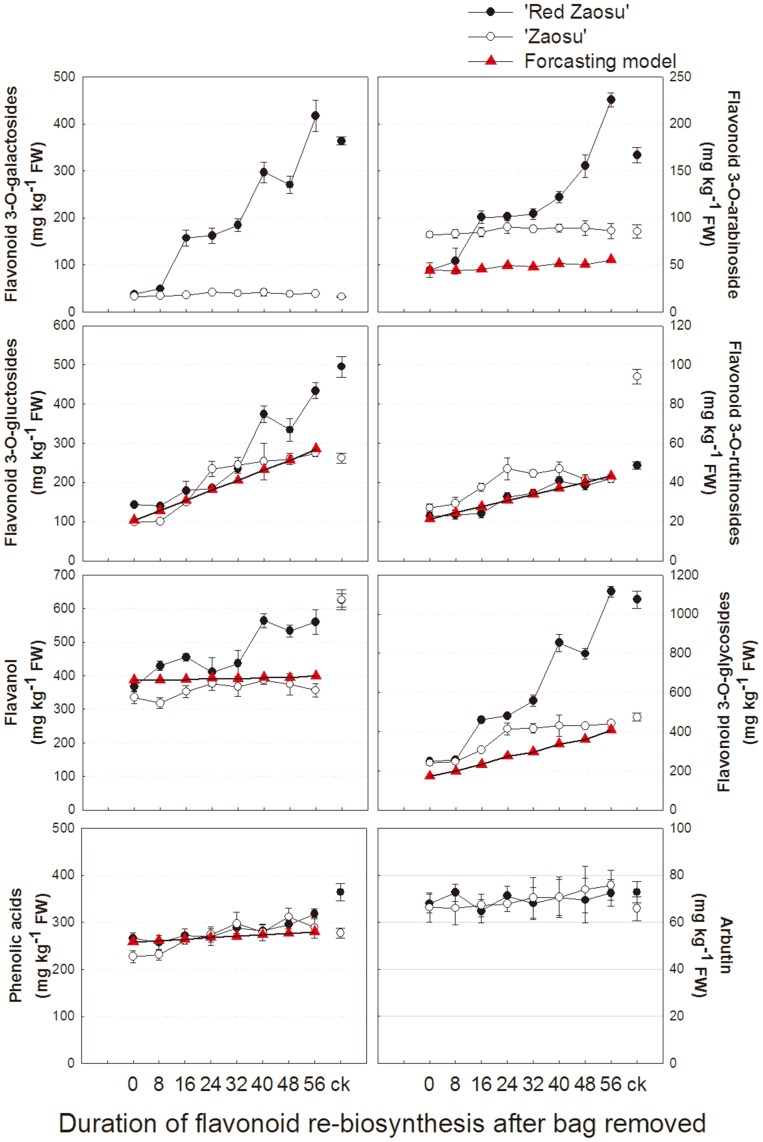
The biosynthesis patterns of flavonoid metabolites and the forecasting model of the fruit peels of ‘Zaosu’ and its mutant ‘Red Zaosu’. The treatments of re-exposed to sunlight for 0 day represented bagged fruits, natural fruits were used as controls. Error bars are SE for 5 replicates.

Arbutin and three major phenolic acid compounds, i.e. gallic acid, chlorogenic acid and caffeic acid, were identified and quantified in the fruit peel of ‘Zaosu’ pear and its red mutant (Fig.3, Table.1). In response to fruit bagging/re-exposure treatments, the concentration of arbutin remained unchanged in the both cultivars (Fig.3, Table.1). The biosynthesis pattern of phenolic acids for the two cultivars did not differ significantly. The levels were slightly inhibited by bagging and gradually recovered after bag removal (Fig.3, Table.1 and Table.2). As a result, ‘Zaosu’ and its red mutant showed similar biosynthesis patterns in upstream flavonoid pathway.

**Table 1:** The phenolic compounds concentrations of the peels of ‘Zaosu’ and its mutant ‘Red Zaosu’ by bagging and re-exposure treatments.

Flavonoid metabolites (mg kg-1 FW)					Flavonoid 3-glycosides				
Treatment	Cultivar	Arbutin	Phenolic acids	Flavonols	Total Flavonoid 3-glycosides	Flavonoid 3-galactosides	Flavonoid 3-gluctosides	Flavonoid 3-rutinosides	Flavonoid 3-arabinoisde
0d	‘Red Zaosu’	67.97±3.89a	266.16±11.52abc	367.09±13.7abc	248.2±12.59a	38.03±2.17a	142.94±5.73ba	22.65±0.7a	44.58±7.75a
	‘Zaosu’	66.25±6.33a	226.96±12.25a	335.21±19.25ab	239.98±10.04a	33.16±3.12a	97.74±5.48a	27±2.1ab	82.09±3.17b
8d	‘Red Zaosu’	72.55±3.72a	256.57±15.36abc	429.33±14.3f	256.45±15.95a	39.26±3.79a	139.67±3.16ba	23.4±2.17a	54.13±13.69a
	‘Zaosu’	65.92±7a	231.5±11.28abc	318.21±15.55abc	246.73±10.41a	34.08±3.21a	100.46±5.63a	29.16±3.31ab	83.04±4.55bc
16d	‘Red Zaosu’	64.72±4.87a	272.27±13.76cd	454.78±11.04cef	460.56±17.52b	157.16±6.94b	178.56±24.49bc	24.17±2.26a	100.68±6.84bcd
	‘Zaosu’	67.18±4.87a	260.75±6.22abc	352.57±17.2abc	306.91±8.82a	35.78±1.32a	148.81±4.75ba	37.54±1.95cd	84.78±5bc
24d	‘Red Zaosu’	71.13±4.1a	268.74±18.13bc	411.02±12.84ef	481.05±7.82bc	162.1±5.86bc	184.91±2.02bc	32.54±2.36bc	101.5±3.08bcd
	‘Zaosu’	67.7±3.13a	314.26±15.95ef	376.89±21.18abc	413.87±31.89b	42.05±2.25a	234.55±18.99cd	46.93±5.58f	90.34±6.62bc
32d	‘Red Zaosu’	67.94±6.73a	288.02±12.01cdef	436.54±19.04g	557.72±29.14c	184.35±13.42c	234.69±11.91cd	34.44±2.01bcd	104.24±5.6cd
	‘Zaosu’	70.44±8.71a	297.1±4.55cdef	366.84±7.41bce	416.41±23.84b	39.55±3.98a	244.08±19.91d	44.46±1.93ef	88.32±3.39bc
40d	‘Red Zaosu’	70.6±8.73a	281.66±13.66cdef	563.94±20.63g	853.31±43.49d	316.88±21.75e	373.75±20.6e	40.68±1.24def	122±6.17d
	‘Zaosu’	70.6±7.65a	278.74±18.04cdef	385.92±12.08abc	430.86±54.33b	41.29±6.84a	253.6±46.98d	46.78±3.81f	89.19±4.23bc
48d	‘Red Zaosu’	69.34±9.53a	295.44±9.47cdef	533.29±17.64g	797.81±26.61d	270.46±9.04d	333.9±8.91e	38.4±2.35cde	155.06±12.33e
	‘Zaosu’	73.93±9.95a	311.75±9.06def	373.89±31.26abc	429.18±14.98b	38.21±3.48a	260.02±14.04d	41.58±2.52def	89.37±7.83bc
56d	‘Red Zaosu’	72.41±5.72a	317.77±10.57f	560.01±36.65def	1115.3±27.23e	416.43±3.22g	431.44±34.56f	41.97±2.04def	225.47±7.58f
	‘Zaosu’	75.76±6.32a	289.11±21.92cdef	356.11±9.51a	441.62±17.63b	39.18±3.24a	274.43±8.57d	41.79±2.01def	86.23±8.03bc
ck	‘Red Zaosu’	72.73±4.61a	364.29±17.71g	624.25±19.65h	1074.04±44.13e	363.44±8.34f	495.28±26.42g	48.61±1.95f	166.71±8.08e
	‘Zaosu’	65.72±5.21a	276.92±10.49cde	625.95±30.49h	474.31±19.94b	32.33±1.88a	262.53±12.76d	93.99±3.57g	85.47±6.95bc

Data were analyzed by Tukey's HSD (honestly significant difference) test (P<0.01), different letters indicate significant difference. ND means not detectable.

**Table 2:** The correlation coefficients of the flavonoid synthesis patterns between the two cultivars.

	Arbutin	Phenolic acids	Flavonoid 3-glycosides	Flavanols
r	-0.04	0.29	0.828**	0.64**
P	0.81	0.08	0.00	0.00

r: Pearson's correlation coefficient.

**means correlation is significant at the 0.01 level (P<0.01, 2-tailed).

Four types of flavanols, i.e. catechin, epicatechin, procyanidin B1 and procyanidin B2, seven types of flavonols, i.e. quercetin 3-galactoside, quercetin 3-gluctoside, quercetin 3-arabinoside, quercetin 3-rutinoside, isrohamnetin 3-galactoside, isrohamnetin 3-gluctoside, isrohamnetin 3-rutinoside and one anthocyanin, i.e. cyanidin 3-galactoside were detected in the pear fruit peels. The concentration of flavanols in the both cultivars was significantly reduced by the bagging treatment. When the fruits were re-exposed to sunlight, flavanols gradually re-accumulated for 40 days in ‘Red Zaosu’ pear, but interestingly, it just slightly re-accumulated for 24 days in ‘Zaosu’ pear (Fig.3, Table. 1 and Table.2). Fruit bagging significantly reduced the concentration of all types of flavonoid 3-glycosides, i.e. anthocyanidin and flavonols in ‘Red Zaosu’. All of these compounds increasingly re-accumulated for 56 days when the fruits were re-exposed to sun light (Fig.3 and Table. 1). In ‘Zaosu’, cyanidin-3-galactoside was not detectable, only rare quecertin-3-galactoside and isrohamnetin-3-galactoside were detected. Two different flavonoid 3-glycosides biosynthesis patterns were found in ‘Zaosu’. In the first pattern, the concentration of flavonol gluctosides and rutinosides was reduced by bagging, and then continuously increased after bag removal, but the concentration of flavonoid 3-galactosides and quecertin 3-arabinoisde were hardly affected by bagging or re-exposure treatment (Fig.3, Table. 1). These results indicated that the main variation of flavonoid profile between ‘Zaosu’ and its red mutant ‘Red Zaosu’ were in the flavonoid 3-glycosides pathway and the flavanol pathway. When the bagged fruits were re-exposed to sunlight, the ability to synthesize flavanols and flavonoid 3-glycosides for ‘Red Zaosu’ could completely recover, but that was much more difficult for ‘Zaosu’. Moreover, two distinct biosynthesis patterns of flavonoid 3-glycosides were found in ‘Zaosu’ pear.

During the fruit developmental process, the concentration of all major flavonoid compounds continuously decreased in ‘Red Zaosu’ and ‘Zaosu’.The main variances of the flavonoid profile between ‘Zaosu’ and ‘Red Zaosu’ were in flavonoid 3-glycosides pathway and flavanol pathway, which were quite similar with that in the bagging and re-exposure treatments. Compared with ‘Red Zaosu’, only flavonoid 3-galactosides were severely suppressed in ‘Zaosu’ during the whole coloring process, which were also consistent with our observation during the re-exposure treatments (Fig.S1).

### Correlations among the biosynthesis patterns of phenolic compounds

Correlations among the synthesis patterns of flavonoid 3-glycosides, flavanols, arbutin and phenolic acids were conducted to define the similarities and the interactions among the synthesis patterns of these flavonoid metabolites. There was no significant difference between the two cultivars in arbutin biosynthesis (p = 0.806) and phenolic biosynthesis (p = 0.084). The synthesis patterns of flavonoid glycosides and flavanols between ‘Red Zaosu’ and ‘Zaosu’ were both correlated but at different levels (positive correlation for flavonoid glycosides, r = 0.828, p<0.01, weak correlation for flavanol, r = 0.636, p<0.01)(Table 2).

A combination analysis of correlation, partial-correlation and multicollinearty tested differences among flavanols, and 4 types of flavonoid 3-glycosides, e.g. flavonoid 3-galactosides, flavonoid 3-gluctosides, flavonoid 3-rutinosides and flavonoid 3-arabinoside in ‘Red Zaosu’ and ‘Zaosu’, respectively. The correlation analysis and multicollinearity test by VIF indicated that flavanol biosynthesis pattern correlated with the biosynthesis patterns of flavonoid 3-gluctosides and flavonoid 3-rutinosides, but the biosynthesis patterns of other flavonoid metabolites were independent, respectively (Table 3). In ‘Red Zaosu’, all biosynthesis patterns of major flavonoid metabolites were correlated, except for arbutin (Table 3). However, in ‘Red Zaosu’, when we used flavonoid 3-galactosides as the control variable in a combination analysis of partial-correlation and multicollinearity test, the correlations coefficients and VIF among other flavonoid severely reduced (Table 3; VIF<10, data not shown), but when we used other components as control variables, the correlation coefficients involved flavonoid 3-galactosides were just slightly affected (Table. 3). This result indicated that the synthesis patterns of flavonoid 3-galactosides was relatively independent, but strongly affected the synthesis patterns of other flavonoid metabolites in the red pear. More interestingly, these results implied that the strongly accumulation of flavonoid 3-galactosides stimulated the biosynthesis of other flavonoid compounds in the red mutant and, therefore, caused systemic variation of flavonoid biosynthesis profiles between ‘Zaosu’ and its red mutant.

**Table 3:** The correlation and partial correlation coefficients of the synthesis patterns of flavonoid 3-glycosides and flavanols in the two cultivars.

	Control Variables											
	without control variables		flavonoid 3-galactosides		flavonoid 3-gluctosides		flavonoid 3-arabinosides		flavonol 3-rutinosides		flavanols	
Correlation pattern	‘Red Zaosu'	‘Zaosu'	‘Red Zaosu'	‘Zaosu'	‘Red Zaosu'	‘Zaosu'	‘Red Zaosu'	‘Zaosu'	‘Red Zaosu'	‘Zaosu'	‘Red Zaosu'	‘Zaosu'
flavonoid 3-galactosides & flavonoid 3-gluctosides	0.96**	0.51**	-	-	-	-	0.75**	0.472**	0.84**	0.7**	0.84**	0.69**
flavonoid 3-galactosides & flavonoid 3-arabinoside	0.94**	0.24	-	-	0.63**	0.07	-	-	0.83**	0.26	0.85**	0.27
flavonoid 3-galactosides & flavonoid 3-rutinosides	0.9**	−0.09	-	-	0.56**	−0.56**	0.7**	−0.12	-	-	0.61**	0.39
flavonoid 3-galactosides & flavanols	0.88**	−0.2	-	-	0.4**	−0.57**	0.68**	−0.23	0.46**	−0.42	-	-
flavonol 3-gluctosides & flavonoid 3-arabinoside	0.9**	0.35	−0.02	0.28	-	-	-	-	0.68**	0.36	0.72**	0.36
flavonoid 3-gluctosides & flavonoid 3-rutinosides	0.86**	0.57**	−0.07	0.73**	-	-	0.49**	0.58**	-	-	0.47**	0.57**
flavonoid 3-gluctosides & flavanols	0.85**	0.45	0.09	0.67	-	-	0.54**	0.45**	0.44**	−0.45**	-	-
flavonoid 3-arabinoside & flavonoid 3-rutinosides	0.81**	0.11	−0.27	0.14	0.18	−0.12	-	-	-	-	0.44**	0.12
flavonoid 3-arabinosides & flavanols	0.78**	0.08	−0.26	0.14	0.06	−0.09	-	-	0.28	0	-	-
flavonol 3-rutinosides & flavanols	0.86**	0.96**	0.32	0.97**	0.47**	0.96**	0.61**	0.96**	-	-	-	-

**means correlation is significant at the 0.01 level (2-tailed).

### Stepwise linear regression model for forecasting flavonoid biosynthesis profiles in ‘Red Zaosu’

To forecast the hypothetical changes of flavonoid profiles when the flavonoid 3-galactosides pathway was inhibited (as well as that in ‘Zaosu’) in ‘Red Zaosu’, regression equations for biosynthesis patterns of major flavonoid compounds, e.g. phenolic acids, flavanols, flavonoid 3-galactosides, flavonoid 3-gluctosides, flavonoid 3-rutinosides, and flavonoid 3-arabinosides, were determined by stepwise linear regression analysis (SLRA), respectively (Table. 4). Suitable independent variables were selected from external cause (re-accumulation duration) and internal causes (major flavonoid compounds). The biosynthesis patterns of flavonoid 3-glycosides, flavanols, and phenolic acids in the forecasting model were similar with that in ‘Zaosu’ (Fig. 3). The results showed that when flavonoid 3-galactosides pathway was inhibited in ‘Red Zaosu’, the biosynthesis patterns of all other major flavonoid compounds were inhibited like ‘Zaosu’, which further proved the hypothesis we mentioned before.

**Table 4:** Regression equation of biosynthesis patterns of major flavonoid compounds.

	adjusted R^2^	regression coefficient	regression constant
flavanols	0.679	0.646*flavonoid 3-galactosides	370.1868405
phenolic acids	0.557	0.115*flavonoid 3-gluctosides	249.3223371
flavonoid 3-arabinoside	0.908	0.631*flavonoid 3-galactosides	29.38
flavonoid 3-rutinosides	0.853	0.389* duration of flavonoid re-biosynthesis	21.38
flavonoid 3-glucosides	0.928	1.289*flavonoid 3-galactosides-1.282*flavonoid 3-arabinosides+3.083*duration of flavonoid re-biosynthesis	103.172

### Expression of key genes in flavonoid biosynthesis and UFGT activity

**Figure 4:**
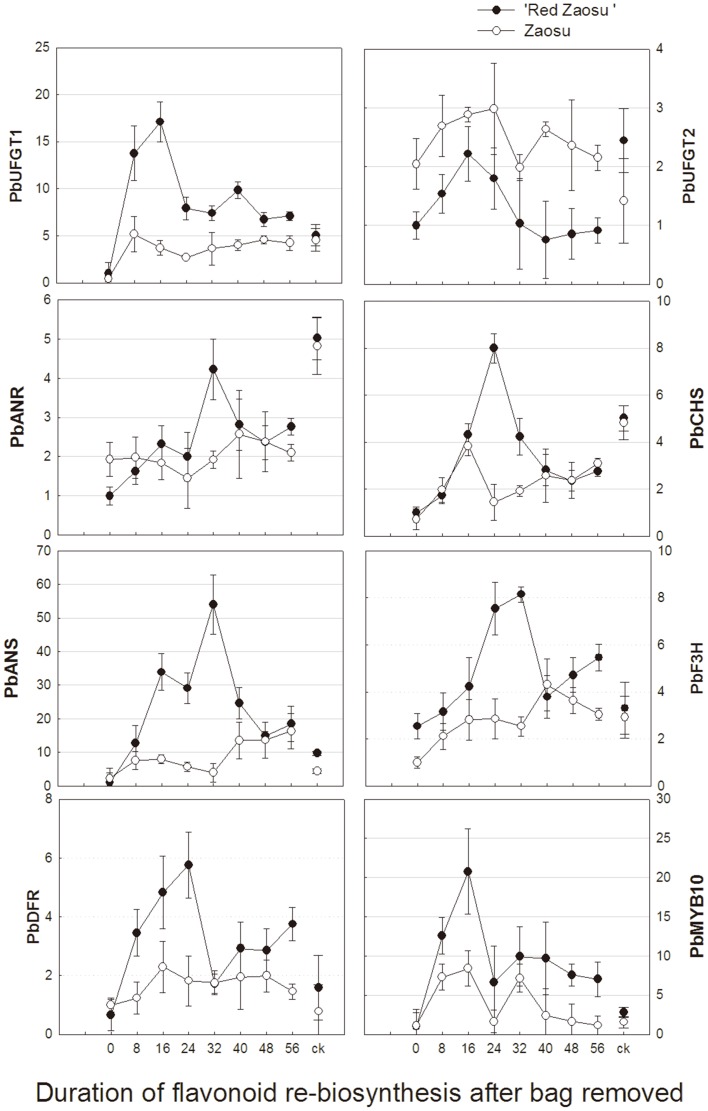
The relative expression patterns of flavonoid structure genes and one transcriptional factor PcMYB10 in the peels of ‘Zaosu’ and its mutant ‘Red Zaosu’ by bagging and re-exposure treatments. The treatments of re-exposed to sunlight for 0 day represented bagged fruits, natural fruits were used as controls. Error bars are SE for 5 replicates.

**Figure 5:**
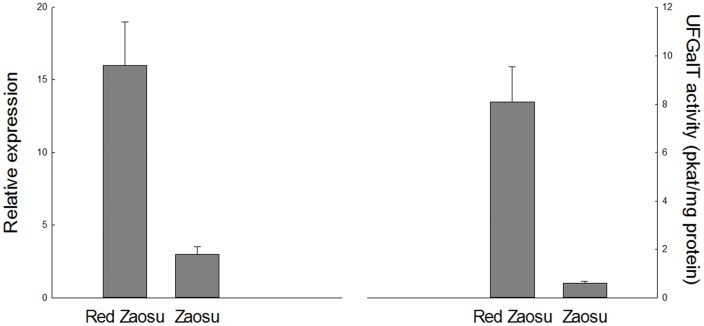
The *PbUFGT1* expression pattern and the enzyme activity of UFGT in the peels of ‘Zaosu’ and its mutant ‘Red Zaosu’. The fruit peel at 16 days after the bags removal was used as the material. Error bars are SE for 5 replicates.

The transcript levels of *PbANR*, *PbANS*, *PbCHS*, *PbF3H*, *PbDFR*, and *PbMYB10* in bagged fruits were significantly lower than that in control fruits and increased to different levels after the bag removal. Compared to its red mutant, ‘Zaosu’ had lower transcript levels but similar synthesis patterns of these genes. Two UFGT genes showed different expression patterns in the both cultivar. *PbUFGT2* showed a quite low transcript level and was not sensitive to bagging and re-exposure treatments in the both cultivars. After the bags were removed, *PbUFGT1* was firstly activated and rapidly increased to a high transcript level, as was *PbMYB10*. Although its expression decreased later, it still remained a quite high level compared with the bagged fruits. The results indicated that *PbUFGT1* and *PbUFGT2* were associated with the two different biosynthesis types of flavonoid 3-glycosides. The expression of other flavonoid biosynthetic genes also increased after the fruits re-exposed to the sunlight, but they reached their maxima later than *PbUFGT1* (Fig.4). Moreover, compared with the high UFGT activity in ‘Red Zaosu’, UFGT was rarely activated in ‘Zaosu’ (Fig.5). These results explained that why the accumulation of flavonoid 3-galactosides was inhibited in ‘Zaosu’ in transcript levels and further demonstrated the strong accumulation of flavonoid 3-galactosides stimulated the biosynthesis of other flavonoid compounds in the red mutant and, therefore, caused systemic variation of flavonoid biosynthesis profiles between ‘Zaosu’ and its red mutant.

### Candidate genes which encoding flavonoid 3-glycosyltransferase

**Figure 6:**
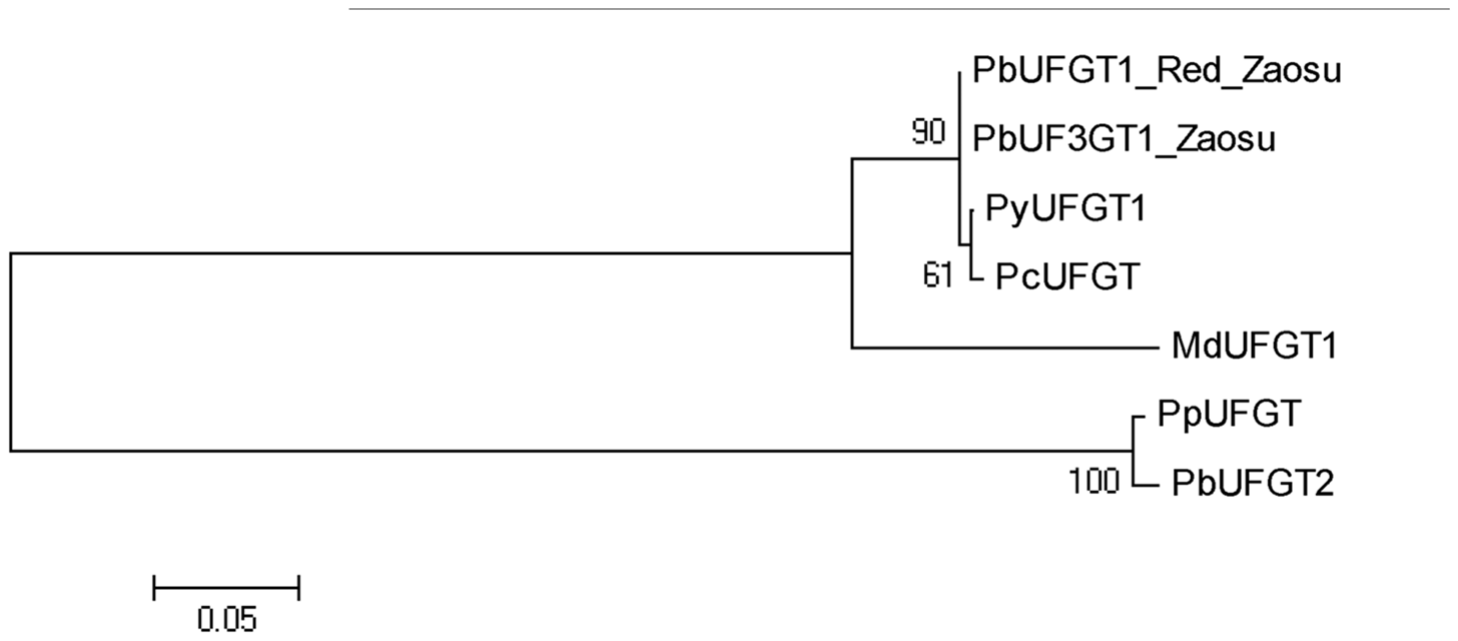
Maximum likelihood phylogeny of UF3GT genes of ‘Zaosu’, ‘Red Zaosu’ and which previous reported in Pyrus and Malus. The tree was produced using MUSCLE and PhyML with the JTT amino acid substitution model.

Two UFGT genes were identified in the Pyrus genome. *PbUFGT1*, same as *PcUFGT* (GenBank accession: JX403956) reported by Wang et al. [9], shared high similarity with *MdUFGT1* (GenBank accession: AF117267); *PbUFGT2* had high homology with *PpUFGT* (GenBank accession: GU390548) reported by Zhang et al. [17] (Fig. 6). The cDNA sequences of *PbUFGT1* containing the complete coding region were isolated from the fruit peels of ‘Zaosu’ and its red mutant, respectively, and showed high homology (at 100%) between ‘Zaosu’ and its red mutant.

## Discussion

Clearly, the bagging treatment lowered most phenolic compound concentrations in the peel of the two cultivars (Figs. 3–4, Table.1). This was consistent with the observation that the activity/expression of key enzymes/genes involved in the flavonoid metabolism pathway could be up-regulated by light irradiations [14, 24 and 25]. Different types of flavonoid 3-glycosides in ‘Zaosu’ had different synthesis patterns. Flavonol arabinosides and flavonol galactosides were hardly affected by bagging and re-exposure treatments; flavonol gluctosides and flavonol rutinosides re-accumulated after the bag removal (Figs. 4–6). Previous studies reported that anthocyanins and flavonols can protect leaves or fruits from photodamage by absorbing visible /UV-B light [2], [3]. The concentration of anthocyanins and flavonols decreased most among flavonoid metabolites by fruit bagging and increased most when the fruits re-exposed to visible and UV-B light [3], [26]–[28]. But in our study, the synthesis pattern of quercetin 3-arabinoside in ‘Zaosu’ showed that it might be not sensitive to light. Its continuous accumulation in the red mutant could be explained: When the bagged ‘Red Zaosu’ fruits were re-exposed to sunlight, quercetin 3-arabinoside was stimulated to continuously accumulate by large amounts of its new synthesized substrate, quecertin, which in response to accumulating other quercetin 3-glycosides, especially quercetin 3-galactosides. Two UFGT genes, i.e. *PbUFGT2* and *PbUFGT1* were isolated from pear genome. Their expressions were totally different and corresponded to synthesis patterns of quercetin 3-arabinoside and other flavonoid glycosides, respectively. In this case, suitable genes should be first identified before further research on its relation with the synthesis pattern of relevant metabolites.

Whether the accumulation of flavanols in fruit peels was independent or dependent of the light conditions to which fruits were exposed were still in dispute in previous studies [3], [29], [30]. Distinct biosynthesis patterns of were found between ‘Zaosu’ and its red mutant, flavanols synthesis was strongly activated when bag removed in the red mutant, but rarely affected by the re-exposure treatments in ‘Zaosu’. The correlation coefficient (r = 0.861) between flavonoid 3-glycosides and flavonols in ‘Red Zaosu’ was higher than that (r = 0.476) in ‘Zaosu’. This variation also could be caused by the biosynthesis flavonoid 3-galactosides in ‘Red Zaosu’. All flavonoid 3-glycosides synthesis patterns were similar in the red mutant. In this multicollinear system, interferences should be erased by a combination analysis of partial correlation and variance inflation factor. The results showed that only flavonoid 3-galactosides made a significant contribution to accumulation of flavanols and other flavonols in ‘Red Zaosu’.

Above all, the main distinctions in flavonoid biosynthesis patterns between ‘Zaosu’ and its red mutant were in the flavonol/anthocyanin pathways and the flavanol pathway. When the fruit bags were removed, the re-accumulation of other flavonoid compounds was probably caused by flavonoid 3-galactosides synthesis in the red mutant. In order to confirm this, the expression patterns of flavonoid structural genes have been determined. *PbUFGT1* and *PbMYB10* were first activated and rapidly increased to a high transcript level in ‘Red Zaosu’, but stayed in a relatively low in ‘Zaosu’. The expression of other flavonoid biosynthetic genes also increased after the fruits were re-exposed to the sunlight, but they reached their maxima later than *PbUFGT1*. This result indicated that the inhibition of *PbUFGT1* maybe the key factor that caused the main distinctions of flavonoid biosynthesis patterns between ‘Zaosu’ and its red mutant, but the similarity of *PbUFGT1* coding sequences in the two cultivars were 100%. Therefore, the ultimate reason could be a transcription factor that can regulate UFGT expression, for example the *MYB10* or another gene in the R2R3-MYB family.

## Supporting Information

Figure S1 **The biosynthesis patterns of flavonoid metabolites in the fruit peels of ‘Zaosu’ and its mutant ‘Red Zaosu’ during the fruit coloring process.** Error bars are SE for 5 replicates.
(TIF)
