# Supplementary figures and images for: Different Biosynthesis Patterns among Flavonoid 3-glycosides with Distinct Effects on Accumulation of Other Flavonoid Metabolites in Pears (Pyrus bretschneideri Rehd.)
Source: PLoS One. 2014 Mar 17;9(3):e91945. doi: 10.1371/journal.pone.0091945 (PMC3956819; doi:10.1371/journal.pone.0091945)

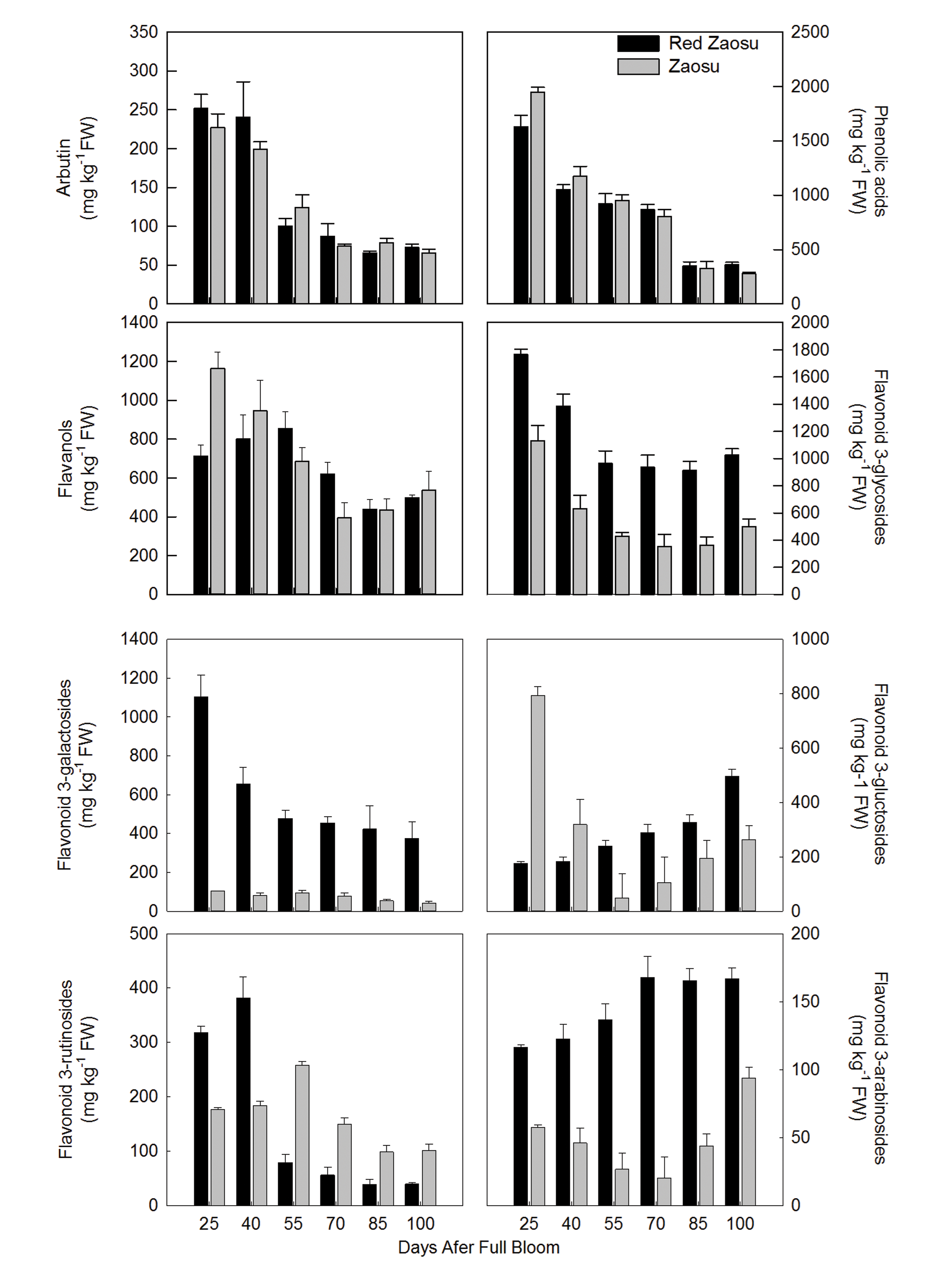

Supplement: Figure S1 — The biosynthesis patterns of flavonoid metabolites in the fruit peels of ‘Zaosu’ and its mutant ‘Red Zaosu’ during the fruit coloring process. Error bars are SE for 5 replicates. (TIF) [file pone.0091945.s001.tif]
